# Evolution of Vertebrate *Adam* Genes; Duplication of Testicular *Adams* from Ancient *Adam9/9-like* Loci

**DOI:** 10.1371/journal.pone.0136281

**Published:** 2015-08-26

**Authors:** Harinath Bahudhanapati, Shashwati Bhattacharya, Shuo Wei

**Affiliations:** Department of Biology, West Virginia University, Morgantown, WV, United States of America; University of Rouen, France, FRANCE

## Abstract

Members of the disintegrin metalloproteinase (ADAM) family have important functions in regulating cell-cell and cell-matrix interactions as well as cell signaling. There are two major types of ADAMs: the somatic ADAMs (sADAMs) that have a significant presence in somatic tissues, and the testicular ADAMs (tADAMs) that are expressed predominantly in the testis. Genes encoding tADAMs can be further divided into two groups: group I (intronless) and group II (intron-containing). To date, *tAdams* have only been reported in placental mammals, and their evolutionary origin and relationship to *sAdams* remain largely unknown. Using phylogenetic and syntenic tools, we analyzed the *Adam* genes in various vertebrates ranging from fishes to placental mammals. Our analyses reveal duplication and loss of some *sAdams* in certain vertebrate species. In particular, there exists an *Adam9-like* gene in non-mammalian vertebrates but not mammals. We also identified putative group I and group II *tAdams* in all amniote species that have been examined. These *tAdam* homologues are more closely related to *Adams 9* and *9-like* than to other *sAdams*. In all amniote species examined, group II *tAdams* lie in close vicinity to *Adam9* and hence likely arose from tandem duplication, whereas group I *tAdams* likely originated through retroposition because of their lack of introns. Clusters of multiple group I *tAdams* are also common, suggesting tandem duplication after retroposition. Therefore, *Adam9/9-like* and some of the derived *tAdam* loci are likely preferred targets for tandem duplication and/or retroposition. Consistent with this hypothesis, we identified a young retroposed gene that duplicated recently from *Adam9* in the opossum. As a result of gene duplication, some *tAdams* were pseudogenized in certain species, whereas others acquired new expression patterns and functions. The rapid duplication of *Adam* genes has a major contribution to the diversity of ADAMs in various vertebrate species.

## Introduction

The first two members of the ADAM family were originally identified as guinea pig sperm-surface antigens that were recognized by the monoclonal antibody PH-30, which strongly inhibits sperm-egg fusion [1]. Sequence analyses of the precursors of both proteins showed that they have a domain organization similar to that of snake venom metalloproteinases, containing a disintegrin domain and a metalloproteinase domain [2,3]. Genes encoding these two proteins, together with several homologues that were cloned from guinea pig and mouse testis, were therefore renamed “*Adams*” (a disintegrin and metalloproteinases) to reflect these important features [4]. It soon became clear that the expression and functions of ADAMs are not limited to the testis. Instead some ADAM family members (*i*.*e*., the sADAMs) have a significant presence in somatic tissues and play important roles in embryonic development [5,6]. To date close to 40 *Adam* genes have been identified in vertebrate and invertebrate animals, and *Adam*-like sequences have also been found in the yeast species *Schizosaccharomyces pombe* [7,8]. Functions of ADAMs in normal development vary from neurogenesis and neural crest development to adipogenesis and myogenesis [6,9,10]. ADAMs have also been implicated in many diseases, including tumors, immune diseases and neurodegenerative diseases [7,8].

A typical ADAM is a type I transmembrane protein, although secreted soluble proteins can also be generated through alternative splicing. The unprocessed form of these transmembrane proteins contains a signal peptide, a pro-domain, a metalloproteinase domain, a disintegrin domain, a cysteine-rich domain, a transmembrane region and a cytoplasmic tail, from N- to C-terminus [7,8]. Only some ADAMs contain the consensus zinc-binding motif (HEXGHXXGXXHD) that is required for metalloproteinase activity, whereas the rest are considered proteolytically inactive. The pro-domain functions as a chaperone to facilitate the folding and secretion of the ADAMs; it also binds to the metalloproteinase domain and inhibits the autocleavage activity of proteolytically active ADAMs. In the processed (“mature”) form of ADAMs, the pro-domain is removed by pro-protein convertases. The mature form of the proteolytically active ADAMs can then act on its substrates, which are mainly cell-surface proteins, to regulate various cellular events including signaling and adhesion. The disintegrin and cysteine-rich domains have been shown to modulate cell-cell and cell-matrix interactions, as well as substrate recognition for the metalloproteinase domain. In particular, the disintegrin domain of several ADAMs can interact specifically with various integrins [7,8]. Finally, the cytoplasmic tail may regulate the functions of extracellular domains through responding to intercellular signals or controlling subcellular localization of ADAMs. It has also been reported that the cytoplasmic tail of ADAM13, an sADAM, is cleaved and translocates into the nucleus, where it regulates target gene expression [11].

The mouse genome contains at least 34 *Adam* genes, while 27 *Adam* loci have been identified to date in the human genome [12,13]. About half of these *Adam* genes are expressed exclusively or predominantly in the testis, and are referred to as *tAdams*. These *tAdams* are further classified into two groups: group I genes (*Adams 1*, *4*, *6*, *20*, *21*, *24–26*, *29*, *30*, *34*, and *36–39*), which have no or few introns; and group II genes (*Adams 2*, *3*, *5*, *18* and *32*), which have multiple introns. None of the group II tADAMs contains the consensus zinc-binding motif in the metalloproteinase domain, and are hence likely non-proteolytic. By contrast, group I tADAMs, except for ADAMs 4, 6 and 29, are predicted to be proteolytically active. It should be noted that some tADAMs, including the presumably proteolytic ADAM1, undergo further processing to remove the metalloproteinase domain during sperm maturation [12,14]. Another unique feature of the tADAMs is that they are often associated with each other and form oligomers on the cell surface [12]. Such complex formation seems to be important for the stability of some tADAMs, as knockout of one tADAM often leads to loss or reduction of another [12,15]. One exception is mouse ADAM24, which has been shown to be present on the sperm surface as a monomer with an intact metalloproteinase domain. This ADAM and several other closely related paralogues that have been found in the mouse genome (ADAMs 25, 26, 34, and 36–39), all predicted to be proteolytically active, are also called “testases” [16,17].

Thus far, *tAdam* genes have only been identified in eutherians, and it is not clear how they originated during evolution and if there are any *tAdams* in other animals. Most of our current knowledge on the functions of tADAMs comes from studies in mice, which show that several *tAdam* genes are important for fertilization in this rodent species. For example, *Adam1a* ^*-/-*^, *Adam2* ^*-/-*^ and *Adam3* ^*-/-*^ male mice are all infertile. Detailed analyses indicate that sperms lacking any of these tADAMs are defective in their migratory activities in the female reproductive tract, as well as in *in vitro* sperm-egg interaction [18–21]. A different phenotype was observed in *Adam24* ^*-/-*^ mice: these mice displayed reduced fertility with high level of polyspermic fertilization [22]. However, some of these genes, such as *Adams 1a* and *3*, appear to have been pseudogenized in the human genome [23,24], suggesting that functions of these genes are species-specific. Therefore, a thorough investigation of the *tAdam* genes in various animal species will not only help us understand how these genes evolved, but also provide important clues on which of these genes may be expressed and functional in individual species. Given the importance of tADAMs in fertilization, the latter information may be of particular interest to veterinary and agricultural research.

In the current study we identified putative group I and group II *tAdams* in all amniote species that have been examined. Phylogenetic and syntenic (gene linkage) analyses suggest that these *tAdams* originally derived from ancient *Adam9*, a somatic *Adam* that is conserved in vertebrates, and/or *Adam9-like*, which we identified here as a close paralogue of *Adam9*. Two mechanisms, tandem duplication and retroposition, were found to underlie the expansion of the *tAdam* genes during vertebrate evolution. These results provide important insight into the evolution of *tAdams*, and form the basis for future functional studies of these important reproductive genes in various vertebrate species.

## Materials and Methods

### Identification of *Adam* genes in various vertebrate species

The sequences of annotated ADAM proteins from humans and mice were BLASTed against the EST databases and genome sequences of other vertebrate species to identify *Adam* genes. Specifically, we performed consistent mining of representative vertebrate species (opossum, chick, *Anolis* lizard, *Xenopus tropicalis* and zebrafish) to understand the broader evolution of the ADAM genes. This was augmented with data obtained from additional species, such as rat, rabbit, pig, dog, platypus, zebra finch, flycatcher, coelacanth, Japanese medaka, cave fish, spotted gar, elephant shark and lamprey, where a more specific understanding of synteny/phylogeny was needed to provide clarity on the data. Potential retroposed *Adam* genes were identified as *Adam* genes with no or few (≤ 2) introns by UCSC Genome Browser BLAT search [25]. To identify the retroposon elements, the *Adam* genes, along with surrounding genome sequences that are 1 kb upstream and downstream, were analyzed with the CENSOR program [26].

### Establishment of orthology with existing *Adam* genes

Three main criteria, phylogeny, synteny, and conservation of exon/intron boundaries, were used to establish the orthology with existing human and mouse *Adams*. Phylogenetic and syntenic analyses were carried out as described below. To determine the exon/intron boundaries of *Adam* genes, deduced amino acid sequences were searched against the UCSC Genome Browser using BLAT [25].

### Phylogenetic analyses

For phylogenetic analyses, deduced amino acid sequences encoded by *Adam* genes from various species (S1 Table) were aligned using MUSCLE (**MU**ltiple **S**equence **C**omparison by **L**og-**E**xpectation; [27]) alignment, and all positions containing gaps were eliminated. Known pseudogenes, such as human *Adams 1*, *3*, *6* and *24*, were not included in the analyses. Phylogenetic trees, including Bayesian, neighbor joining and maximum likelihood trees, were built to assess the evolutionary relationship among ADAMs as described below.

The Bayesian analyses were conducted with MrBayes version 3.2.2 [28]. Each analysis was run for 3,000,000 iterations with six chains to achieve a mean standard deviation less than 0.01. Trees were sampled every 100 iterations. The final tree was visualized with FigTree v4.1.0 [29].

Neighbor joining and maximum likelihood trees were generated by using the corresponding algorithms included in the MEGA 6.0 software [30]. For neighbor joining trees, the bootstrap consensus trees inferred from 1000 replicates were used to represent the evolutionary history of the taxa analyzed. The evolutionary distances were computed using the numbers of amino acid substitutions per site with the Poisson correction method [31]. The Maximum Likelihood method is based on the JTT matrix-based model [32]. Initial trees were obtained by applying the neighbor joining and BioNJ algorithms to a matrix of pairwise distances using the JTT model, and then the topology was selected with superior log likelihood value.

### Syntenic analyses

For syntenic analyses, an individual *Adam* gene was entered into the search box of Genomicus [33], Metazome [34], and NCBI Map Viewer to identify surrounding loci that are evolutionarily conserved and can serve as syntenic anchor genes. Data from these websites were combined to generate the syntenic maps. Loss of an *Adam* gene in a specific species was validated by searching the sequences of nearby genomic regions that include the syntenic anchor genes.

## Results and Discussion

### ADAMs in various animal species

A previous study by Huxley-Jones *et al*. identified 4 and 22 *Adam* genes in *Ciona* and zebrafish, respectively, and we have also reported the presence of 15 homologues of mammalian *sAdam* genes in *Xenopus tropicalis* [35,36]. In all published analyses on ADAMs from various invertebrate and vertebrate species, ADAMs 10 and 17 stand out phylogenetically as a separate clade from the rest of sADAMs [35–38]. These two ADAMs are highly conserved throughout animal evolution, with orthologues present in invertebrates such as *C*. *elegans*, *Drosophila* and *Ciona* [35,37]. One exception is sea urchin, where ADAM10 seems to be absent [38]. There is another ADAM in sea urchin and two in *Drosophila* and *Ciona*, which do not belong to the ADAM10/17 clade. These ADAMs cluster with the rest of mammalian sADAMs, but no distinct orthology could be established [35].

**Fig 1:**
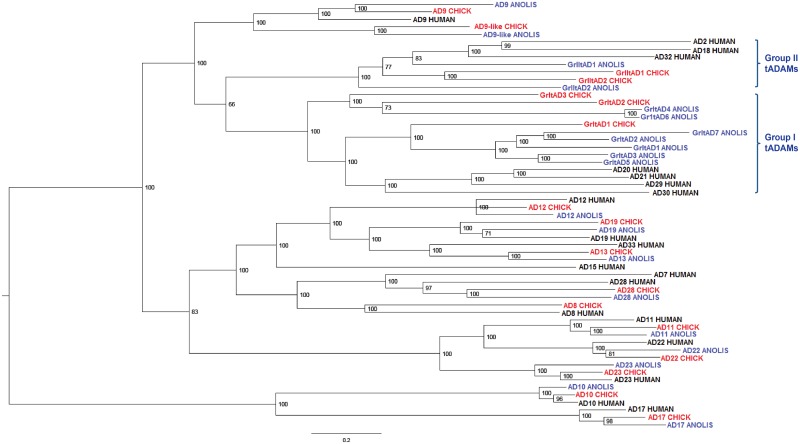
Bayesian tree of ADAMs from *Anolis*, chick and human. Sequences of ADAMs from *Anolis* (blue), chick (red) and human (black) were aligned, and Bayesian trees were generated as described in Materials and Methods. The numbers shown are Bayesian posterior probability values.

In contrast to the few sADAMs in invertebrates, orthologues of most mammalian sADAMs, including ADAMs 9, 10, 11, 12, 15, 17, 19, 22, 23, 28 and 33, have been identified in both zebrafish and *Xenopus* [35,36], suggesting an expansion of *sAdam* genes during early vertebrate evolution. However, these published studies failed to identify an orthologue of ADAM7 in zebrafish and *Xenopus*, and ADAM8 seems to be missing in *Xenopus* [35,36]. Moreover, no tADAM orthologue has been reported in any non-eutherian species [35–38]. To understand the evolution of *Adam* genes and, particularly, the origins of *tAdams*, we performed a search for *Adam* genes in various vertebrate species, including *X*. *tropicalis*, *Anolis*, chick, opossum, mouse and human. Phylogenetic trees of deduced protein sequences encoded by these genes were generated by three methods: Bayesian (Fig 1 and S1 Fig), neighbor joining (S2 Fig), and maximum likelihood (S3 Fig); the results obtained with different methods agree well with each other. We also carried out syntenic analyses for these *Adam* genes using the online comparative genomics tools such as Genomicus and Metazome [33]. The results are summarized below.

### Absence of several *sAdams* in certain vertebrate species

**Fig 2:**
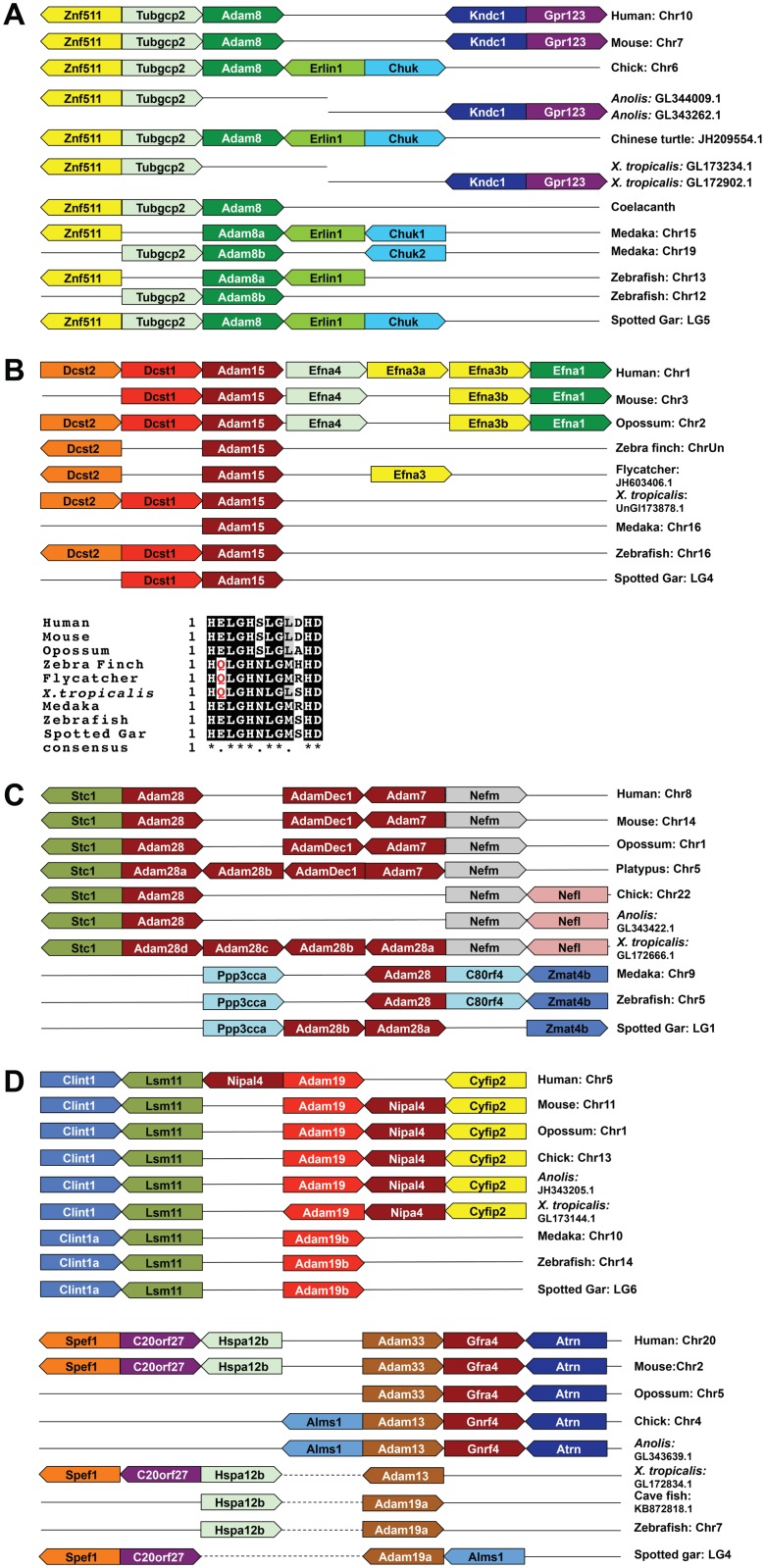
Syntenic analyses of certain sADAMs. A. Absence of *Adam8* in the *X*. *tropicalis* and *Anolis* genomes. B. Loss of ADAM15 gene or protease activity in non-mammalian tetrapods. Sequences of ADAM15 proteins from various vertebrate species were aligned with ClustalW and colored using BoxShade, and the glutamine found in certain species instead of the glutamic acid in the conserved zinc-binding motif is highlighted in red. C. Absence of *Adam7* and *Adamdec1* in non-mammalian vertebrates. D. Syntenic analyses showing that fish *Adam19b* is the orthologue of tetrapod *Adam19*, whereas fish *Adam19a* is the orthologue of tetrapod *Adam13/33*. Syntenic maps were generated as described in Materials and Methods. Dashed lines indicate that genes are several loci away from each other (same below).

We showed previously that the genomic region surrounding *Adam8* underwent substantial reorganization during vertebrate evolution, likely resulting in the loss of *Adam8* in *X*. *tropicalis* [36]. Similarly, we could not identify *Adam8* in the *Anolis* genome, although an *Adam8* orthologue does exist in Chinese softshell turtle, another reptile species, and in coelacanth, one of the closest living fish relatives of tetrapods (Fig 2A; [39]). A single *Adam8* gene was found in all mammalian and avian species that have been examined, and there are two copies of *Adam8* in the genomes of several teleost fish species (Fig 2A and [35]), likely as a result of teleost genome duplication (TGD; [40,41]). This is supported by the presence of a single *Adam8* gene in the spotted gar (Fig 2A), which diverged from teleosts before the TGD and has a genome organization more similar to that of humans than teleosts [42]. Our syntenic analysis further shows that most non-mammalian vertebrates share a similar genome structure in this region, but *X*. *tropicalis* and *Anolis* have undergone chromosome reorganization that likely resulted in loss of *Adam8* (Fig 2A).

Another notable loss of gene or gene function lies in the *Adam15* locus. The mammalian *Adam15* encodes an active metalloproteinase, whose activity has been demonstrated using several substrates [43,44]. However, we have predicted that both *X*. *tropicalis* and *X*. *laevis* ADAM15 proteins are non-proteolytic, as they contain a glutamine instead of the glutamic acid that is found in the consensus zinc-binding motif [36]. In the current study we were unable to find an *Adam15* orthologue in the *Anolis* or chicken genome. *Adam15* does exist in the zebra finch and flycatcher, two avian species, but the deduced protein sequences show that both contain the E to Q substitution that was previously found in frogs (Fig 2B). A separate report also suggests that *Adam15* is lost in the chick but not zebra finch [45]. On the other hand, fishes seem to have a well-conserved *Adam15* gene encoding a proteolytic enzyme (Fig 2B), suggesting that the loss of ADAM15 or its proteolytic activity is probably specific for non-mammalian tetrapods.

On human chromosome 8p12, genes encoding ADAM28, ADAMDEC1 (a soluble ADAM-like protein) and ADAM7 form a cluster, likely as a result of tandem duplication [46]. Similar clustering of these three *Adams* was found in other mammalian species including marsupials and monotremes, except that in platypus *Adam28* was further duplicated to give rise to two genes in tandem (Fig 2C). Four and two tandem copies of *Adam28* exist in *X*. *tropicalis* and spotted gar, respectively, but no definitive orthologue of *Adam7* or *Adamdec1* could be identified (Fig 2C and [36]). We were also unable to identify either *Adam7* or *Adamdec1* in any other non-mammalian vertebrate genomes that have been investigated, including those of aves, reptiles and fishes. Instead, in most of these non-mammalian vertebrates, a single *Adam28* locus is present in this region (Fig 2C and data not shown). Therefore, the duplication of *Adam28* in the spotted gar, *X*. *tropicalis* and platypus seems to be independent events. These data also suggest that *Adam7* and *Adamdec1* were duplicated from *Adam28*, probably only in mammals. Subsequently, ADAM7 and ADAMDEC1 underwent neo- or subfunctionalization, as well as the loss of ADAM7 protease activity and truncation of ADAMDEC1 that removed part of the disintegrin domain and all the sequence C-terminal to it [36,46].

Our previous phylogenetic and syntenic analyses indicate that *Xenopus* ADAM13, which plays an important role in neural crest induction and migration as well as eye formation, is an orthologue of mammalian ADAM33 [11,36,47,48]. ADAM13/33 also exists in all avian and reptile species that have been examined (Fig 2D and data not shown). However, Huxley-Jones *et al*. reported the presence of two *Adam19* loci (*Adam19a* and *Adam19b*) but not *Adam13/*33 in zebrafish [35], raising the question whether *Adam13/33* only exists in tetrapods. The two *Adam19* loci in zebrafish are unlikely the result of TGD, as both are also present in the spotted gar (Fig 2D). A syntenic comparison with other vertebrate species reveals that fish *Adam19b* is the orthologue of tetrapod *Adam19*, whereas fish *Adam19a* is in fact the orthologue of tetrapod *Adam13/33* (Fig 2D). This is consistent with our previous phylogenetic analysis, which showed that zebrafish ADAM19b clusters with tetrapod ADAM19 and zebrafish ADAM19a clusters with tetrapod ADAM13/33 with high confidence [36]. Thus ADAM13/33 is conserved from fishes to mammals.

### Presence of an *Adam9*-like gene in non-mammalian vertebrates

**Fig 3:**
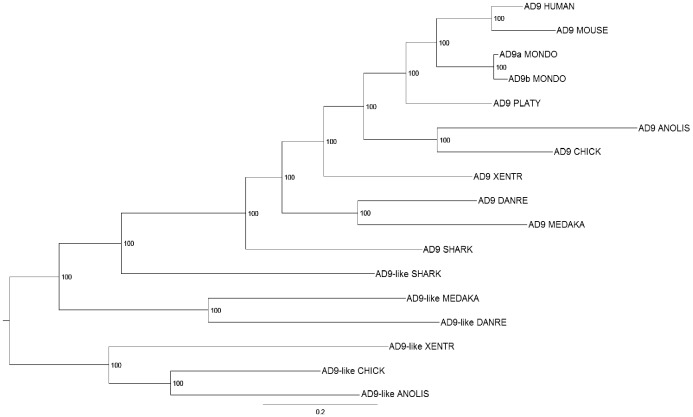
Bayesian trees of ADAMs 9 and 9-like from various vertebrate species. MONDO, opossum; PLATY, platypus; XENTR, *X*. *tropicalis*; and DANRE, zebrafish. Note that opossum *Adam9b* is predicted to be a pseudogene with a frameshift.

**Fig 4:**
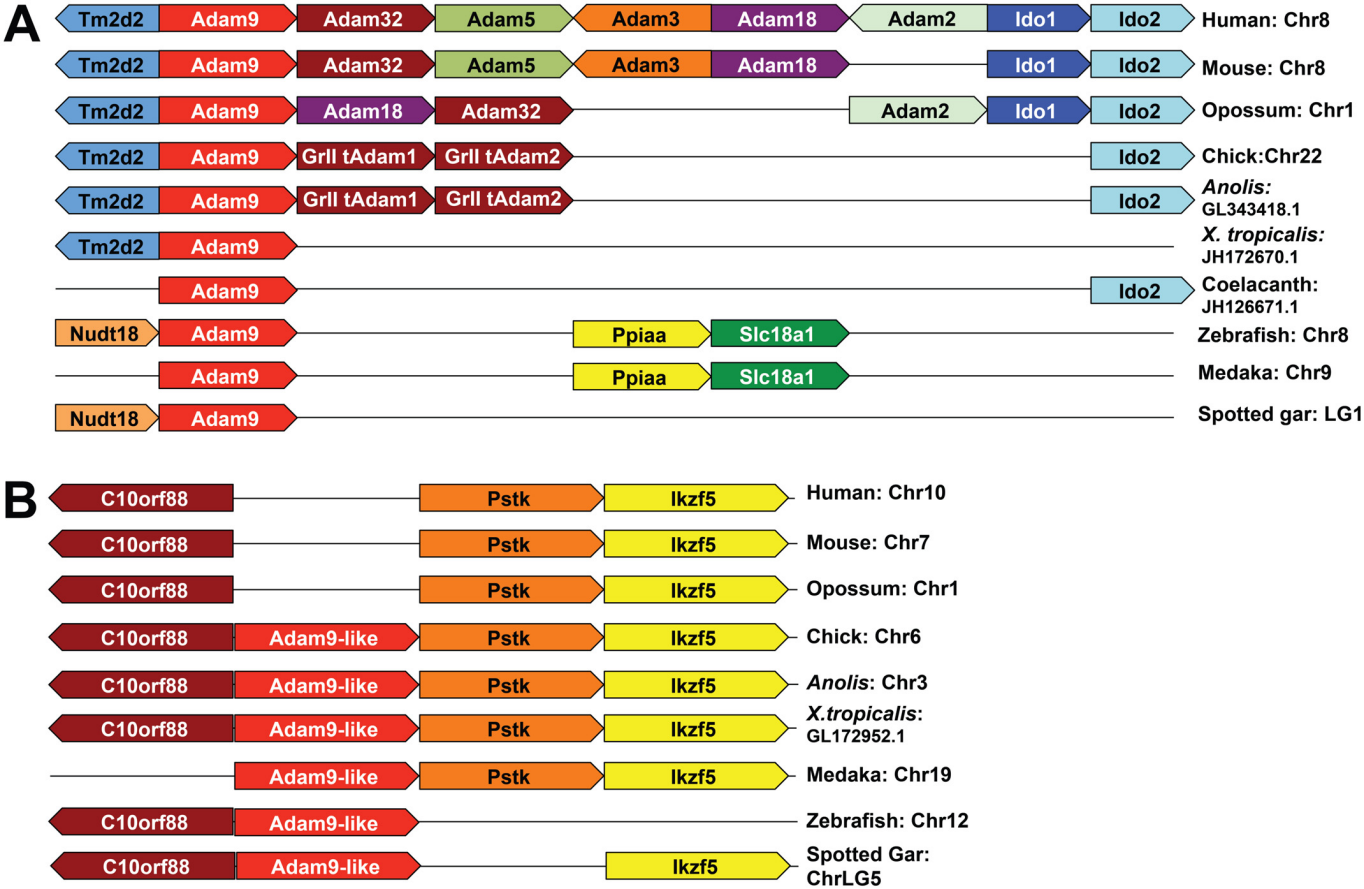
Syntenic analyses of ADAMs 9 (A) and 9-like (B).

ADAM9 is an sADAM that plays important roles in tumor progression, including metastasis and angiogenesis [49]. Although *Adam9* ^*-/-*^ mice do not show any apparent developmental defects, they develop retinal degeneration during aging, and null mutations in *Adam9* have been linked to early onset cone-rod dystrophy in humans [50]. Previous studies did not identify an *Adam9* orthologue in invertebrates such as *Drosophila* and *Ciona*, but a partial sequence with high homology to mammalian *Adam9* was reported in zebrafish [35]. We searched the Ensembl database and found the full-length sequence of this gene; phylogenetic analysis indicates that it is the zebrafish orthologue of *Adam9* (Fig 3). An *Adam9* gene also exists in other fishes, including the cartilage fish elephant shark, as well as all other vertebrate species that have been examined (Figs 3 and 4A).

We also identified an *Adam9-like* gene in non-mammalian vertebrates, including fishes, amphibians, reptiles and aves; however, this gene seems to have been lost in mammals (Figs 3 and 4B). The predicted protein sequences encoded by the *Adam9-like* genes from various species are more similar to ADAM9 than to other sADAMs (Fig 1, S1A, S2A-S2C and S3A-S3C Figs). Similar to *Adam9*, the presence of *Adam9-like* can also be traced to the elephant shark (Fig 3), but neither *Adam9* nor *9-like* could be identified in the jawless fish lamprey. Thus both *Adams 9* and *9-like* likely emerged during early vertebrate evolution. However, we were unable to determine which gene emerged first, due to the lack of an available genome sequence between the lamprey and elephant shark.

### Group I and group II tADAMs exist in non-eutherian amniotes and form an ADAM subfamily with ADAMs 9 and 9-like

In an attempt to find potential *tAdam* genes in non-eutherian vertebrates, we identified close homologues of human and mouse group I and group II *tAdams* in *Anolis*, chick and opossum (Fig 1 and S1B Fig), but not in *X*. *tropicalis* or any of the fish species. Group I and group II tADAMs from various vertebrates form distinct clades, and both clades cluster with ADAMs 9 and 9-like (Fig 1, S1B, S1C, S2B-S2F and S3B-S3F Figs). Although no close homologue of any mammalian *tAdam* has been identified in the *X*. *tropicalis* genome [36], there is an *Adam16* (also called *xMdc*) gene that has been reported to be expressed exclusively in the testis of *Xenopus laevis* [51]. Interestingly, ADAM16 also clusters with ADAMs 9 and 9-like (S1A, S2A and S3A Figs), and hence may be a tADAM that evolved separately in frogs and related species. Our findings that group I and group II tADAMs belong to the same ADAM subfamily that also includes ADAMs 9 and 9-like, and that ADAMs 9 and 9-like likely emerged earlier than both groups of tADAMs during vertebrate evolution, suggest that group I and group II tADAMs evolved from ancient ADAM9 and/or 9-like. In the literature, the clade of *Adams 7* and *28* are sometimes classified together with the *tAdams* as “reproductive” *Adams*, as *Adams 7* and *28* are predominantly expressed in the mouse epididymis [12]. However, information available in the UniGene database (UID 158438) shows that human *Adam28* is expressed in a wide variety of somatic tissues, and our phylogenetic analyses strongly indicate that ADAMs 7 and 28 do not belong to the subfamily that includes ADAMs 9, 9-like and tADAMs. Thus ADAMs 7 and 28 were not included in the subsequent analyses.

### Origins of group II and group I *tAdam* genes: evidence for tandem duplication and retroposition, respectively

Syntenic comparison of group II *tAdams* shows a unique feature: in all amniote species that were examined, group II *tAdams* cluster together in a genomic region that also includes *Adam9* (Fig 4A). The only exception is rodent *Adam2*, which is localized in a different region. For example, in the mouse genome *Adam2* lies in chromosome 14 and *Adam9* in chromosome 8, whereas in the rat genome *Adam2* is localized in chromosome 15 and *Adam9* in chromosome 16. No other *Adam* gene was found in the close vicinity of *Adam9* in the *X*. *tropicalis* or zebrafish genome. By contrast, two group II *tAdam* genes lie next to *Adam9* in the *Anolis* and chicken genomes, whereas three, four and five were found in this region in the opossum, mouse and human genomes, respectively (Fig 4A). These results suggest that tandem duplication gave rise to group II *tAdam* genes, and that an ancient *Adam9* was likely the parental gene that generated the very first group II *tAdam* during evolution.

Unlike group II *tAdams*, which have multiple introns, group I *tAdams* have few or no introns. Because lack of introns is characteristic of retroposed genes, it has been speculated that group I *tAdams* derived from retroposition [12]. However, there is no published evidence that supports this speculation, and it was not clear from which gene(s) group I *tAdams* were retroposed. As discussed above, our phylogenetic and syntenic analyses suggest that group I *tAdams* share a common ancestor with group II *tAdams* as well as *Adams 9* and *9-like*. Thus the parental gene that gave rise to a specific group I *tAdam* could be *Adam9*, *9-like*, a group II *tAdam*, or another group I *tAdam*. Because nearly all of these group I *tAdams* emerged a long time ago, they have lost the signatures of retroposed genes (other than the lack of introns) that may serve as evidence for the retroposition events they underwent. We therefore started out to search for young retroposed genes that derived from the *Adam9/9-like/tAdam* subfamily, in which the characteristic features of retroposition may still be well preserved.

**Fig 5:**
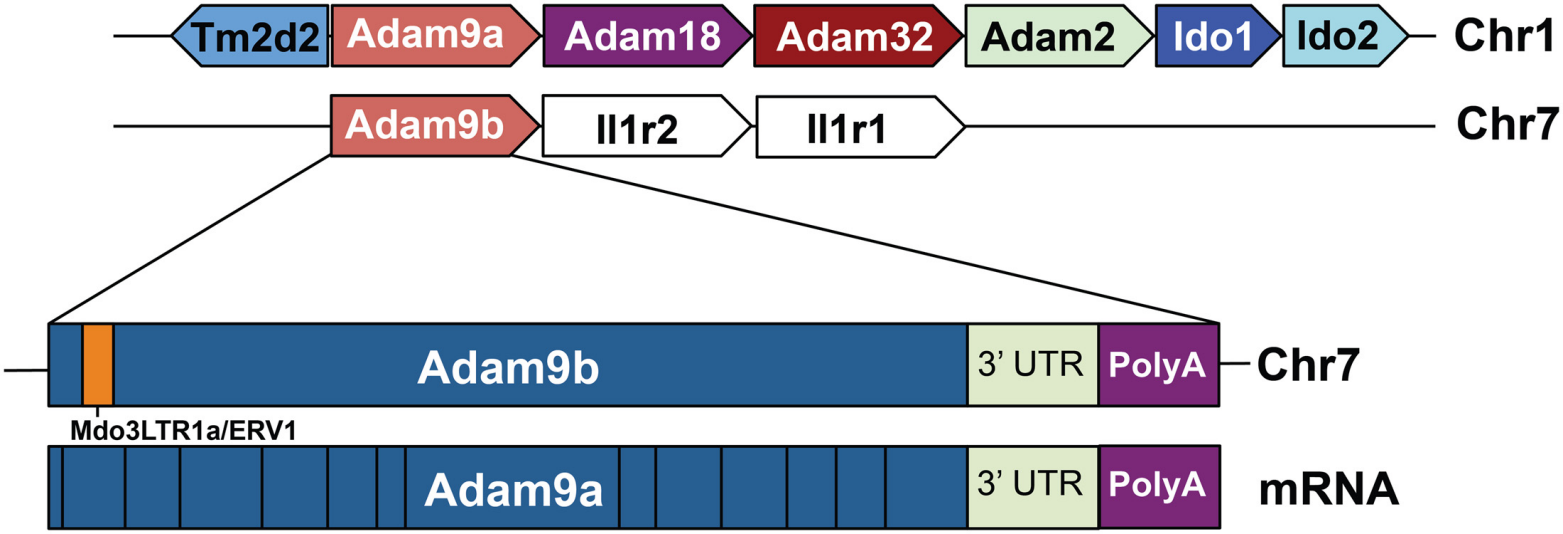
A recent duplication of *Adam9* through retroposition. Locations of *Adams 9a* and *9b* in the opossum genome, as well as the gene structure of *Adam9b*, are shown. Structure of the *Adam9a* mRNA is also shown for comparison, with black vertical lines representing splice sites (not to scale). The *Adam9b* pseudogene contains a polyA sequence, and the only intron was identified as an opossum retroposon (Mdo3LTR1a/ERV1), which was likely inserted after the original retroposition event that generated *Adam9b*. This insertion caused a frameshift.

In the opossum genome, we identified an additional copy of *Adam9* that has only one intron (Fig 5). This gene, which we named *Adam9b*, is ~99% identical to the other copy (*Adam9a*) in nucleotide sequence. The *Adam9a* gene contains 24 introns and conserved exon/intron boundaries that are found in *Adam9* genes of other vertebrate species (data not shown), and is hence the opossum orthologue of *Adam9*. The lack of introns and very high sequence homology to *Adam9a* suggest that *Adam9b* was likely retroposed recently from *Adam9a*. This is further supported by the presence of a polyA tail in the *Adam9b* gene (Fig 5). Because the marsupial genomes contain a high percentage of retroposed elements [52], we tested if other *sAdams* have also been retroposed. No potential retroposed copy of any other *sAdams* could be identified in the opossum genome, suggesting that *Adam9* may be a preferred target for retroposition. A recent study shows that the retroposon element L1 has preference for certain transcripts, presumably those that are expressed in the germ cells, where L1 activity is high [53]. In line with this finding, transcripts of *Adam9* are expressed in the testis of *Xenopus*, mouse and human ([51] and S2 Table).

### Further expansion of group I *tAdam* genes through tandem duplication

**Fig 6:**
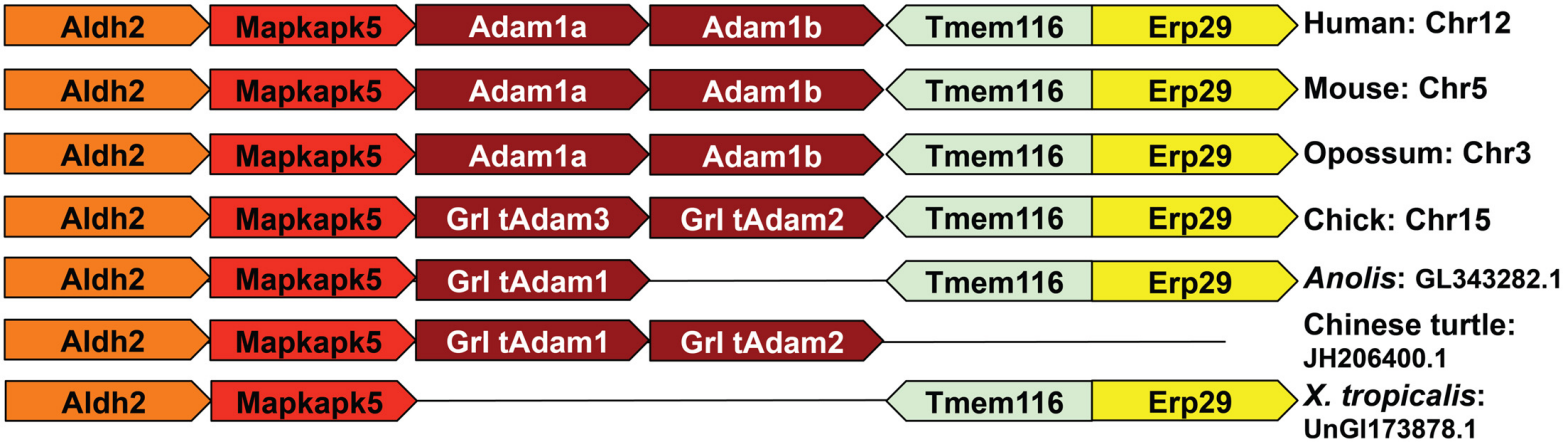
Tandem duplication of *Adam1* in most amniote species.

Clusters of group I *tAdam* genes were found in various amniote species, suggesting tandem duplication after retroposition. One example is the duplication of the *Adam1* locus (Fig 6). Although gene organization in the surrounding loci is conserved from frogs to mammals, no *Adam* gene was found in the *X*. *tropicalis* genome in this genomic region. By contrast, there is one copy of *Adam1* in the *Anolis* genome and two tandem copies in the chicken, opossum, mouse and human genomes (Fig 6). However, no strict orthology could be established between mammalian and chicken *Adam1* genes. One possible explanation for these observations is that *Adam1* originated before mammals diverged from reptiles and aves, and was duplicated separately in mammals and aves. Alternatively, *Adam1* may have been duplicated before the divergence of mammals, but one copy was lost in certain species such as the *Anolis*. In line with the latter model, two tandem copies of *Adam1* were also identified in the Chinese softshell turtle (Fig 6).

**Fig 7:**
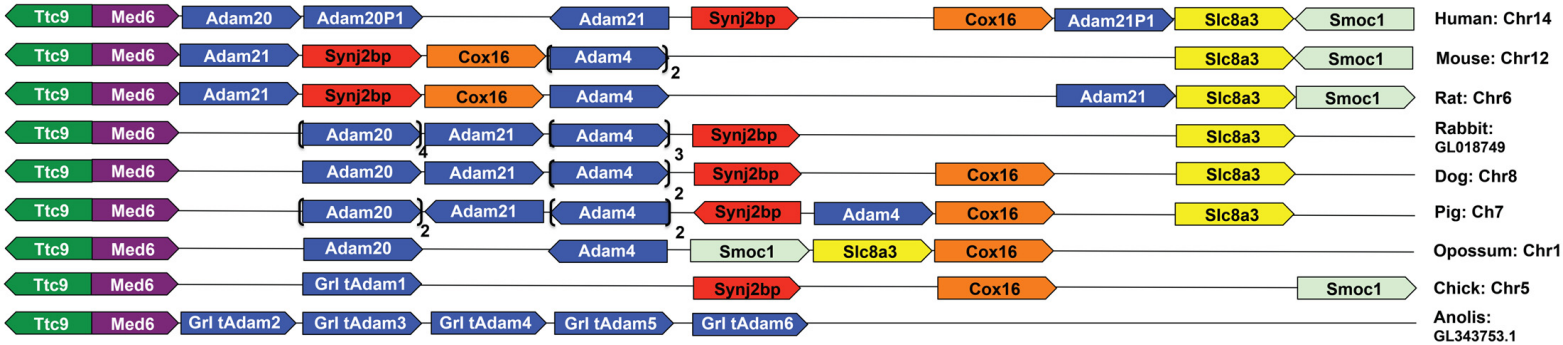
Tandem duplication of *Adams 4*, *20* and *21* in mammals. Multiple copies of *Adams 4* and *20* are shown in brackets with copy numbers indicated.

Tandem duplication was also found in the genomic region that includes *Adams 4*, *20* and *21*. The total number of *Adam4/20/21* loci in this region varies from species to species among amniotes, ranging from one in the chick to eight in the rabbit (Fig 7). Accompanying gene duplication, occasional gene loss also occurred. For example, *Adam20* seems to be absent in rodents but not in most other eutherians (Fig 7).

**Fig 8:**
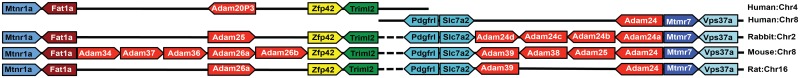
Localization of testase genes in euarchontoglire genomes. ADAMs 24, 25, 26a, 26b, 34, and 36–39 are testases 1, 2, 3a, 3b, 4, and 6–9, respectively. Both human *Adam20P3* and *Adam24* are pseudogenes, and *Adam20P3* is a close paralogue of *Adam20*.

The testases are a clade of proteolytically active group I tADAMs [16,17]. Nine testase genes have been cloned or predicted in the mouse genome, but it is not clear if orthologues of these genes exist elsewhere. We were unable to find any testase genes in any vertebrate species other than euarchontoglires; therefore the testases are probably specific for these mammals. On mouse chromosome 8, all nine testase genes cluster into two sub-regions that are separated by ~10 genes. Similar organizations were found on corresponding chromosomes of rat and rabbit, two other glire species, except that there are fewer testase genes than in the mouse genome (Fig 8). In the human genome, this region seems to have undergone chromosomal reorganization, and the two sub-regions were found in two separate chromosomes. There is one *tAdam* locus in each sub-region, but both loci appear to have been pseudogenized (Fig 8). Therefore, these two loci likely existed in early euarchontoglires, but the large-scale duplication of testase genes that occurred in the mouse, rat and rabbit likely happened after the divergence of glires.

### The fate of *tAdam* genes after duplication

A common consequence of gene duplication is the loss of function of duplicated genes. The HUGO Gene Nomenclature Committee website listed 11 human *Adam* pseudogenes, all of which are *tAdams*, including both group I and group II genes [13]. The pseudogenization of these *tAdams*, including group I *tAdams* that likely derived from retroposition, is mainly caused by premature stop codons rather than lack of transcription (data not shown). Retroposed genes were thought to be non-functional because they were not expected to carry any regulatory elements required for transcription [54]. However, it has been shown that many retroposed genes in the human genome are actively transcribed, probably by localizing closely to other genes and utilizing the regulatory elements and/or open chromatin structures of these neighboring genes [55]. Direct inheritance of basic promoters from parental genes has also been reported for retroposed genes [56]. Interestingly, a large fraction of retroposed genes, along with some other duplicated genes, is expressed initially in the testis [54,55]. This has been attributed to the hypertranscriptional state of the meiotic and post-meiotic spermatogenic cells, which facilitates transcription of otherwise silenced genes, such as those derived from retroposition or other means of gene duplication [57,58]. Therefore, it is not surprising that the group I *tAdam* genes, which likely derived from retroposition, are predominantly expressed in the testis.

As discussed above, recently duplicated genes are often expressed in the testis. These actively expressed genes may acquire new functions, and will be maintained if the new functions provide advantages during selection. These genes may also expand their expression/functions to other tissues [54]. Consistent with this theory, the mouse *Adam2* promoter was reported to bind Mrf/Gm98, a transcriptional activator in the central nervous system [59], and neuroblasts from ADAM2 knockout mice show defects in their migration toward the olfactory bulb [60]. A search against the UniGene database revealed that other group II *tAdams* are also expressed in some somatic tissues in mice and humans (S2 Table). Therefore, the expression and functions of *tAdam* genes are not limited to the testis.

## Conclusions

In this study we show that certain *Adam* genes were frequently lost or duplicated during vertebrate evolution. We also report here the existence of homologues of mouse and human *tAdams* in various non-eutherian amniotes. Our phylogenetic and syntenic analyses suggest that *tAdams* form a subfamily with *Adam9* as well as an *Adam9-like* gene, which seems to be present in non-mammalian vertebrates only. Group II *tAdams* likely originated from an ancient *Adam9* gene through tandem duplication, whereas group I *tAdams* seem to have derived initially from retroposition and also undergone extensive tandem duplication in amniotes. One striking example is the clustering of the whole testase gene clade, which contains 9 group I *tAdams* in mice, in a single chromosomal region. Most of the *tAdam* loci maintain the distinct features of duplicated genes, such as tandem localization and lack of introns, providing excellent examples for studying gene duplication. It would be of interest in the future to determine if the non-eutherian *tAdam* genes that we identified in this study are indeed expressed predominantly in the testis. However, some of these genes may have expanded their expression to other tissues and acquired new functions.

## Supporting Information

S1 Fig Bayesian trees of ADAMs from *Xenopus*, opossum and mouse. (DOCX)

S2 Fig Neighbor joining trees of ADAMs from various vertebrate species. (DOCX)

S3 Fig Maximum likelihood trees of ADAMs from various vertebrate species. (DOCX)

S1 Table Accession numbers of ADAM proteins used in phylogenetic analyses. (XLSX)

S2 Table Expression patterns of *Adam9* and Group II *tAdams* in mice and humans. (DOCX)
