# Supplementary material for: Evolution of Vertebrate Adam Genes; Duplication of Testicular Adams from Ancient Adam9/9-like Loci
Source: PLoS One. 2015 Aug 26;10(8):e0136281. doi: 10.1371/journal.pone.0136281 (PMC4550289; doi:10.1371/journal.pone.0136281)
Supplement: S1 Fig — (DOCX) [file pone.0136281.s001.docx]

**
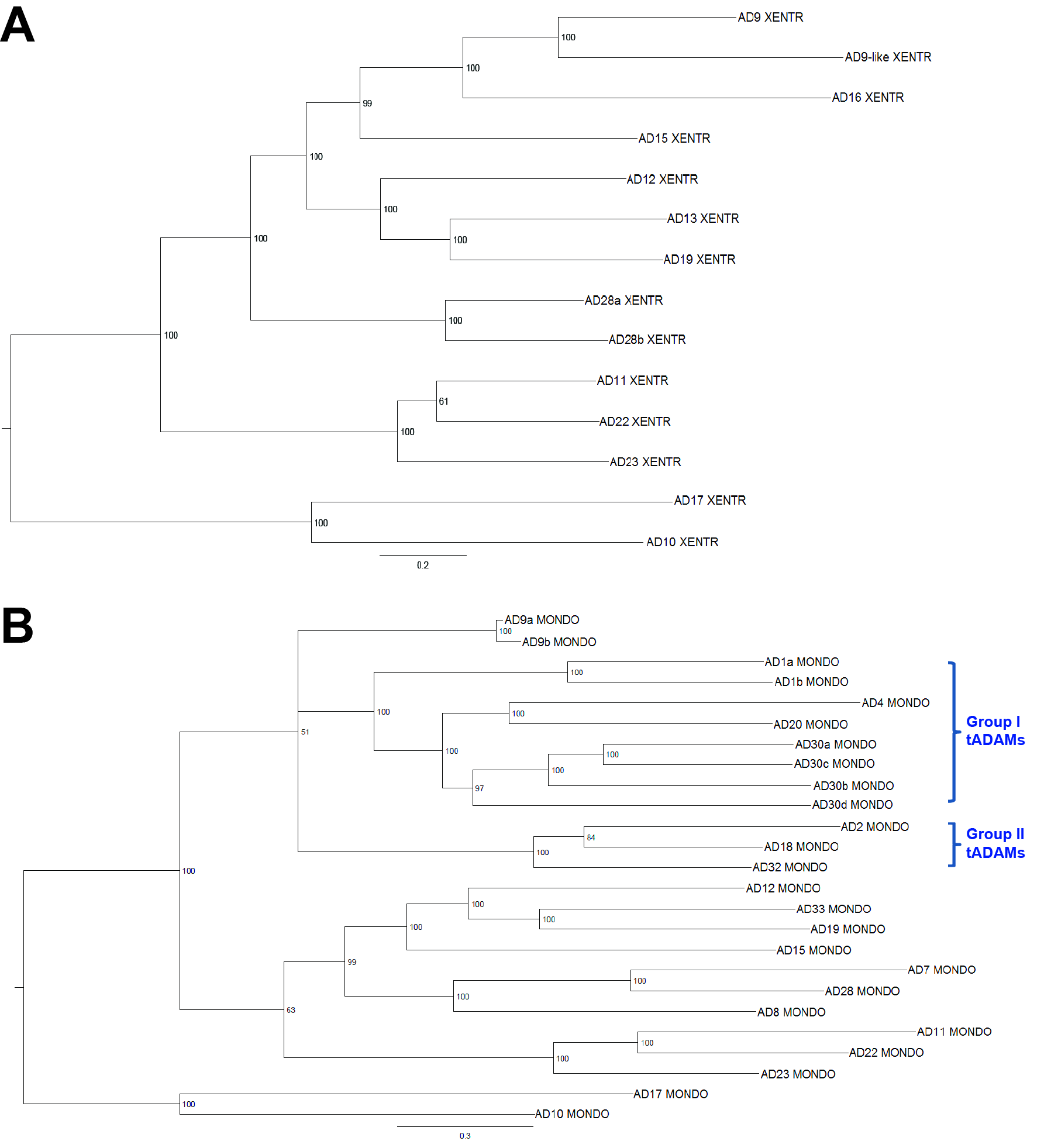
**

**
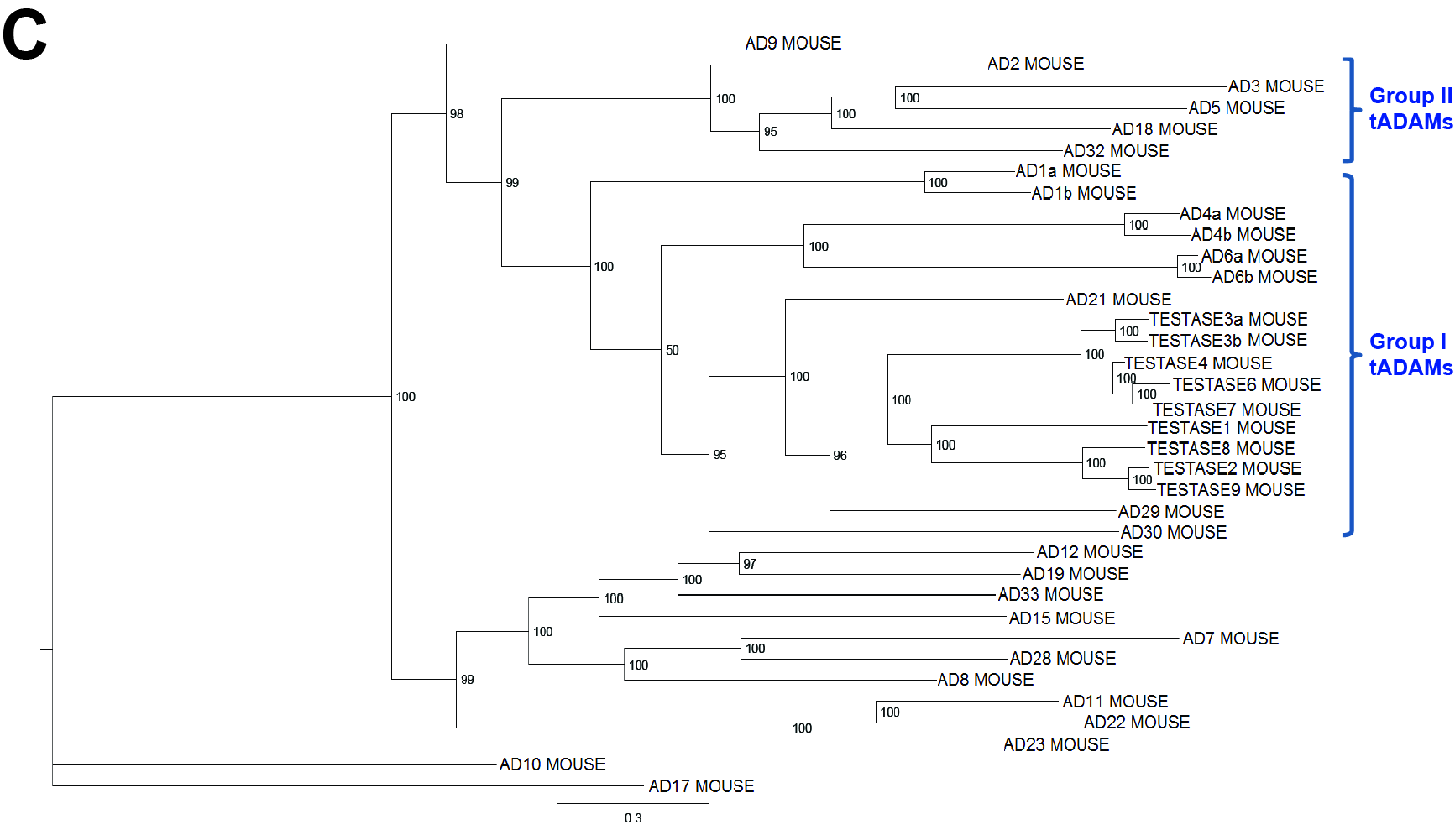
**

**Figure S1. Bayesian trees of ADAMs from *Xenopus*, opossum and mouse.** Sequences of ADAMs from *X. tropicalis* (XENTR; A), opossum (MONDO; B), and mouse (C) were aligned, and Bayesian trees were generated as described in Materials and Methods. The numbers shown are Bayesian posterior probability values. Mouse testases 1, 2, 3a, 3b, 4, and 6-9 are ADAMs 24, 25, 26a, 26b, 34, and 36-39, respectively.
