# Supplementary material for: Evolution of Vertebrate Adam Genes; Duplication of Testicular Adams from Ancient Adam9/9-like Loci
Source: PLoS One. 2015 Aug 26;10(8):e0136281. doi: 10.1371/journal.pone.0136281 (PMC4550289; doi:10.1371/journal.pone.0136281)
Supplement: S2 Fig — (DOCX) [file pone.0136281.s002.docx]

**
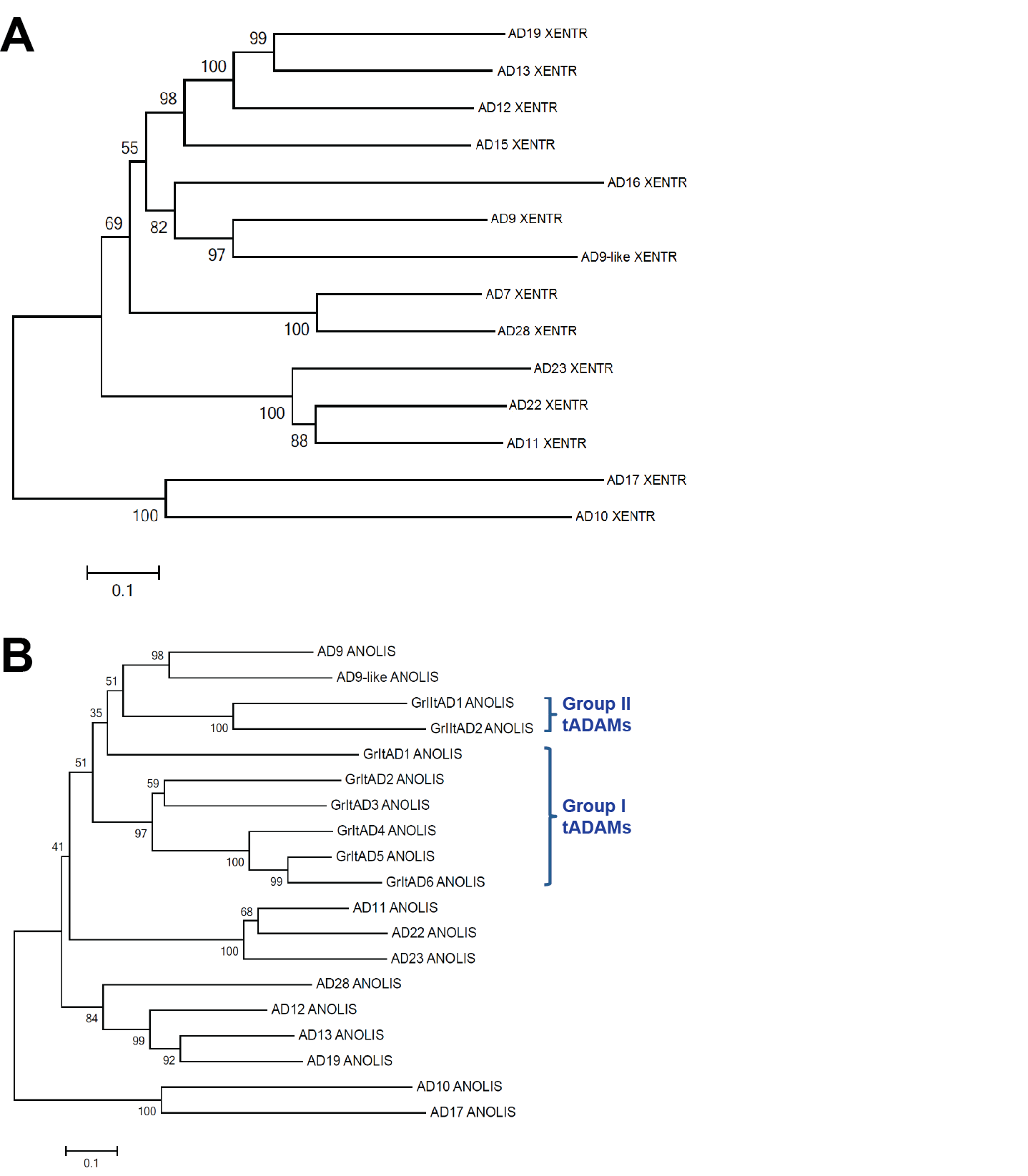
**

**
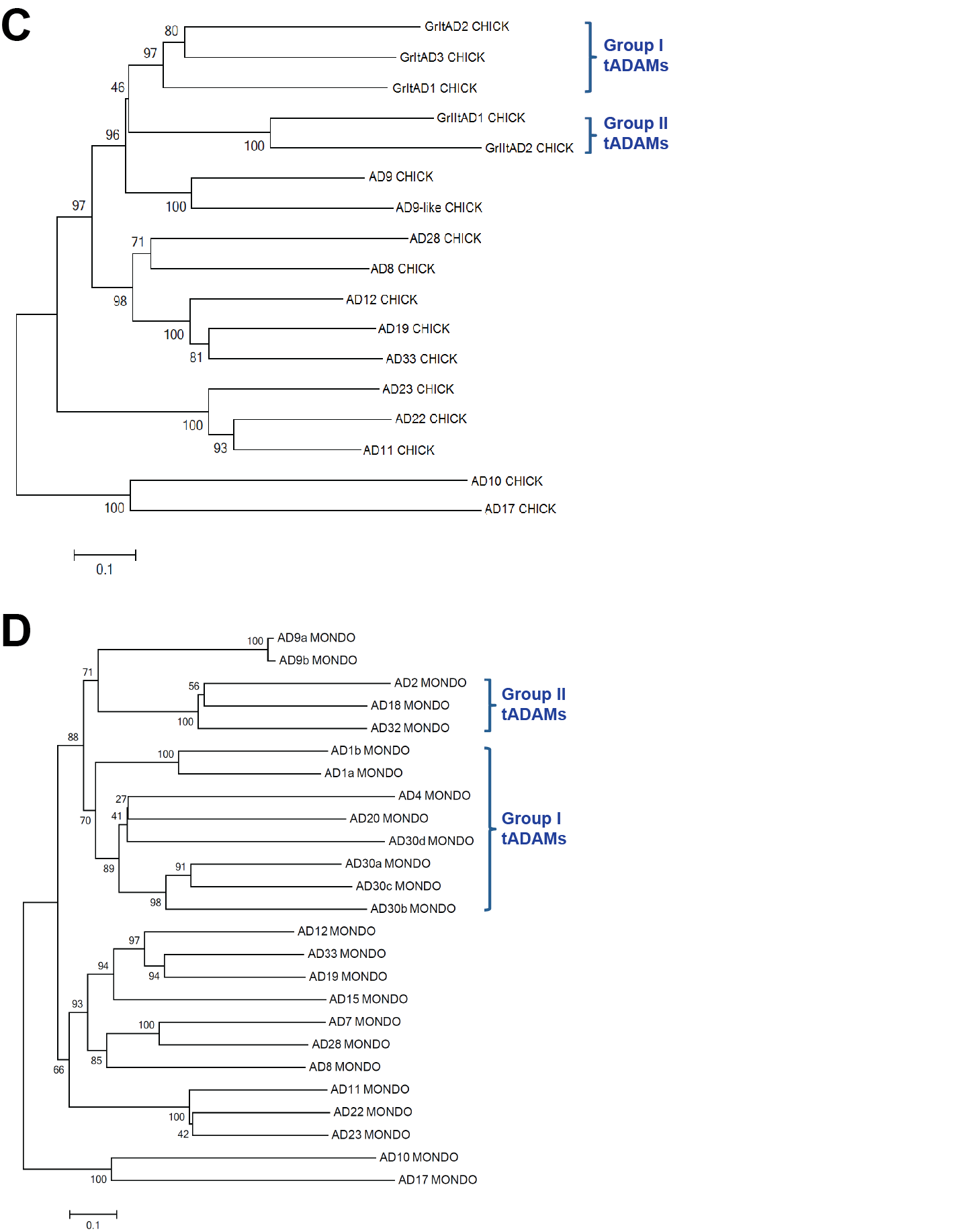
**

**
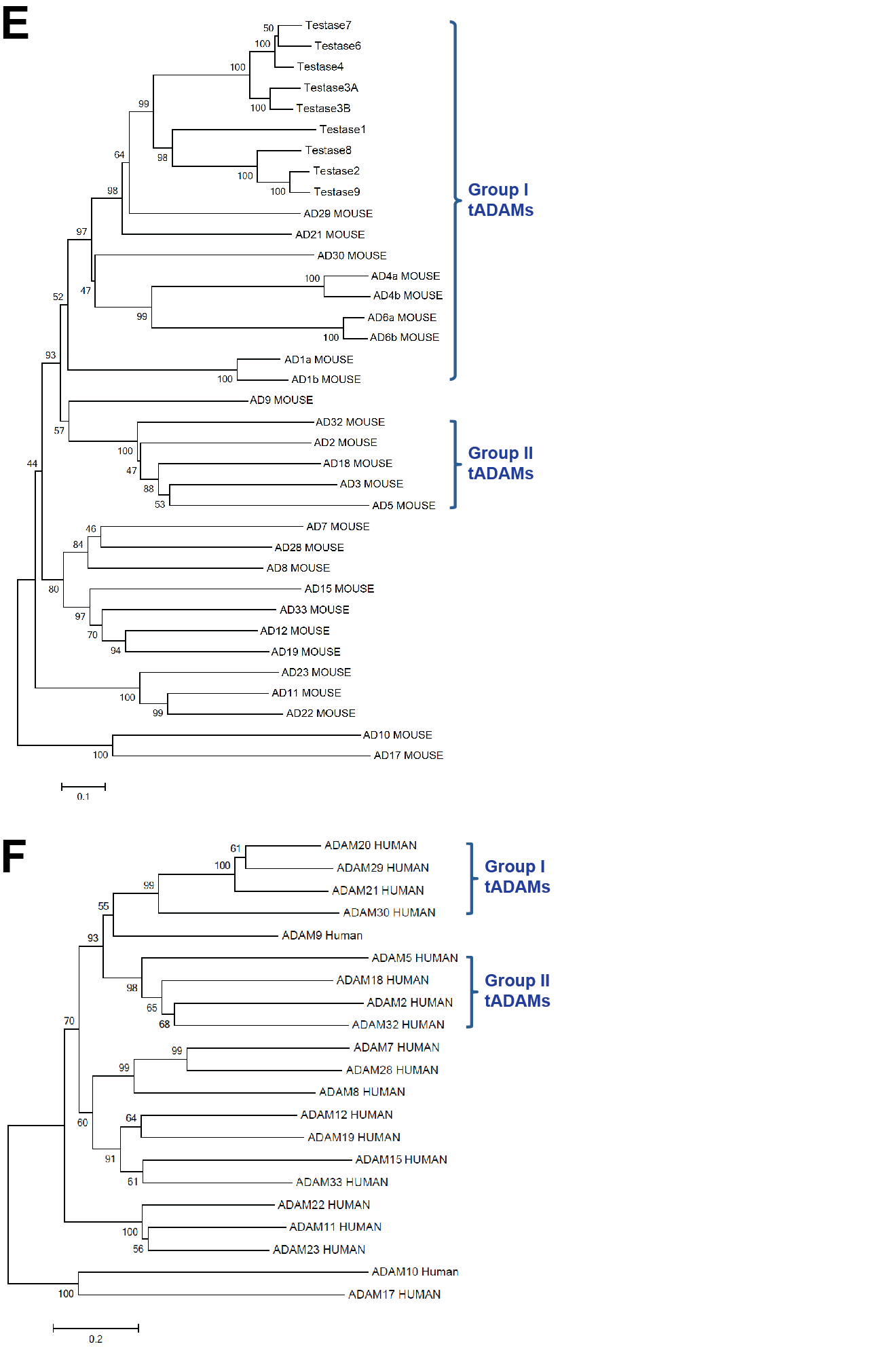
**

**Figure S2. Neighbor joining trees of ADAMs from various vertebrate species.** Sequences of ADAMs from *X. tropicalis* (XENTR; A), anolis (B), chick (C), opossum (MONDO; D), mouse (E) and human (F) were aligned, and neighbor joining trees were generated as described in Materials and Methods. The numbers shown are bootstrap values.
