# Supplementary material for: Evolution of Vertebrate Adam Genes; Duplication of Testicular Adams from Ancient Adam9/9-like Loci
Source: PLoS One. 2015 Aug 26;10(8):e0136281. doi: 10.1371/journal.pone.0136281 (PMC4550289; doi:10.1371/journal.pone.0136281)
Supplement: S2 Table — (DOCX) [file pone.0136281.s005.docx]

**Table S2: Expression patterns of *Adam9* and Group II *tAdams* in mice and humans**

| **Gene** | **Mouse** | | | **Human** | | |
| --- | --- | --- | --- | --- | --- | --- |
| *Adam9* | **Tissue** | **Transcript per million** | **Gene EST/Total EST in pool** | **Tissue** | **Transcript per million** | **Gene EST/Total EST in pool** |
|  | adipose tissue | 649 | 1/1540 | adipose tissue | 77 | 1/12866 |
|  | bladder | 429 | 7/16283 | adrenal gland | 60 | 2/32940 |
|  | bone | 440 | 15/34066 | ascites | 75 | 3/39834 |
|  | bone marrow | 29 | 4/136333 | bladder | 133 | 4/29860 |
|  | brain | 33 | 16/475384 | brain | 21 | 24/1092688 |
|  | connective tissue | 504 | 10/19807 | connective tissue | 120 | 18/149072 |
|  | embryonic tissue | 112 | 76/677554 | embryonic tissue | 56 | 12/212896 |
|  | epididymis | 322 | 1/3101 | nerve | 128 | 2/15535 |
|  | eye | 80 | 15/185387 | eye | 23 | 5/208840 |
|  | heart | 36 | 2/54558 | heart | 44 | 4/89524 |
|  | mammary gland | 141 | 43/303048 |  |  |  |
|  | testis | 32 | 4/121820 | testis | 27 | 12/435204 |
|  | uterus | 291 | 2/6855 | uterus | 21 | 5/232093 |
|  | sympathetic ganglion | 400 | 4/9986 | vascular | 271 | 14/51649 |
| *Adam5* | connective tissue | 50 | 1/19807 | connective tissue | 46 | 7/149072 |
|  | testis | 582 | 71/121820 | testis | 68 | 30/435204 |
|  | eye | 5 | 1/185387 |  |  |  |
| *Adam3* | spleen | 86 | 8/92417 | brain | 3 | 4/1092688 |
|  | testis | 1264 | 154/121820 | testis | 117 | 51/435204 |
|  |  |  |  |  |  |  |
| *Adam18* | testis | 82 | 10/121820 | testis | 64 | 28/435204 |
|  |  |  |  | bladder | 33 | 1/29860 |
|  |  |  |  | connective tissue | 46 | 7/149072 |
|  |  |  |  | embryonic tissue | 9 | 2/212896 |
| *Adam32* | embryonic tissue | 7 | 5/677554 | adipose tissue | 77 | 1/12866 |
|  | testis | 213 | 26/121820 | testis | 124 | 54/435204 |
|  |  |  |  | connective tissue | 114 | 17/149072 |
|  |  |  |  | brain | 3 | 4/1092688 |
|  |  |  |  | kidney | 4 | 1/210778 |
| *Adam2* | thymus | 2 | 1/121153 | brain | 1 | 2/1092688 |
|  | lungs | 10 | 1/99799 | connective tissue | 20 | 3/149072 |
|  | testis | 147 | 36/435204 | testis | 82 | 36/435204 |
|  |  |  |  | prostate | 10 | 2/189536 |

Tissue expression patterns were obtained from NCBI’s UniGene website.
